# Case Report: Traumatic cardiac arrest due to pericardial tamponade: successful pericardiocentesis with a Shaldon catheter

**DOI:** 10.3389/fped.2024.1383061

**Published:** 2024-05-10

**Authors:** Maximilian Schilling, Alexandre Serra, Christian Skrabal, Harald Ehrhardt, Bettina Jungwirth, Sebastian Schmid

**Affiliations:** ^1^ Department of Anesthesiology and Intensive Care, Faculty of Medicine, University of Ulm, Ulm, Germany; ^2^ Division of Paediatric Surgery, Department of Surgery, University Medical Center Ulm, Ulm, Germany; ^3^ Klinik für Kinderchirurgie, Klinik St. Hedwig, Krankenhaus der Barmherzigen Brüder, Regensburg, Germany; ^4^ Department of Cardiac Surgery, University Medical Center Ulm, Ulm, Germany; ^5^ Division of Neonatology and Pediatric Intensive Care Medicine, University Medical Center Ulm, Ulm, Germany

**Keywords:** traumatic cardiac arrest, emergency ultrasonography, sanguineous pericardial tamponade, pericardiocentesis, Shaldon adult catheter, pericardial drainage, PoCUS, penetrating trauma

## Abstract

In this report, we describe the successful resuscitation of a 4-year-old child who suffered a traumatic cardiac arrest during a routine procedure in the operating room. The diagnosis of a sanguineous pericardial tamponade was made by emergency ultrasonography. Consecutive subxiphoid pericardiocentesis with an adult Shaldon catheter led to return of spontaneous circulation. Subsequent thoracotomy and surgical suturing definitively stopped the bleeding from the right ventricle. The combined expertise of all perioperative disciplines was decisive for the patient's survival.

## Learning points

(1) Sanguineous pericardial tamponade can be relieved by pericardiocentesis through a Shaldon adult catheter.
(2) Training in sonographic diagnosis and invasive therapy of traumatic cardiac arrest is essential for patient survival.
(3) With adequate resources, management of complications is possible even in children.

## Introduction

Intra-hospital traumatic cardiac arrest (TCA) is an extremely rare event in childhood (1). We report a case of a 4-year-old child with TCA who could be successfully treated by pericardiocentesis, blood transfusion and median sternotomy for hemostasis.

## Case report

A 4-year-old normally developed 16 kg child with a relapse of acute lymphoblastic leukemia (ALL) required a repeat tunneled central line (Hickman) catheterization for further therapy. The child was anesthetized and monitored as usual.

There had been two previous Hickman catheter dislocations in the child's history, which had required surgical correction and reimplantation. Preoperative sonographic evaluation of the vascular situation showed that both internal jugular veins were thrombosed. A preoperative CT scan had not been indicated, but in retrospect could have influenced the implantation. Anticoagulation was not administered because the child had thrombocytopenia and nosebleeds, which required a nasal tamponade. Due to the thrombosed jugular veins, the chosen access was the right subclavian vein, which was exceptionally challenging to catheterize in this child even under radiological control. Accordingly, it was necessary to change from a 9 French sheath to a smaller 7 French sheath during the procedure.

**Figure 1:**
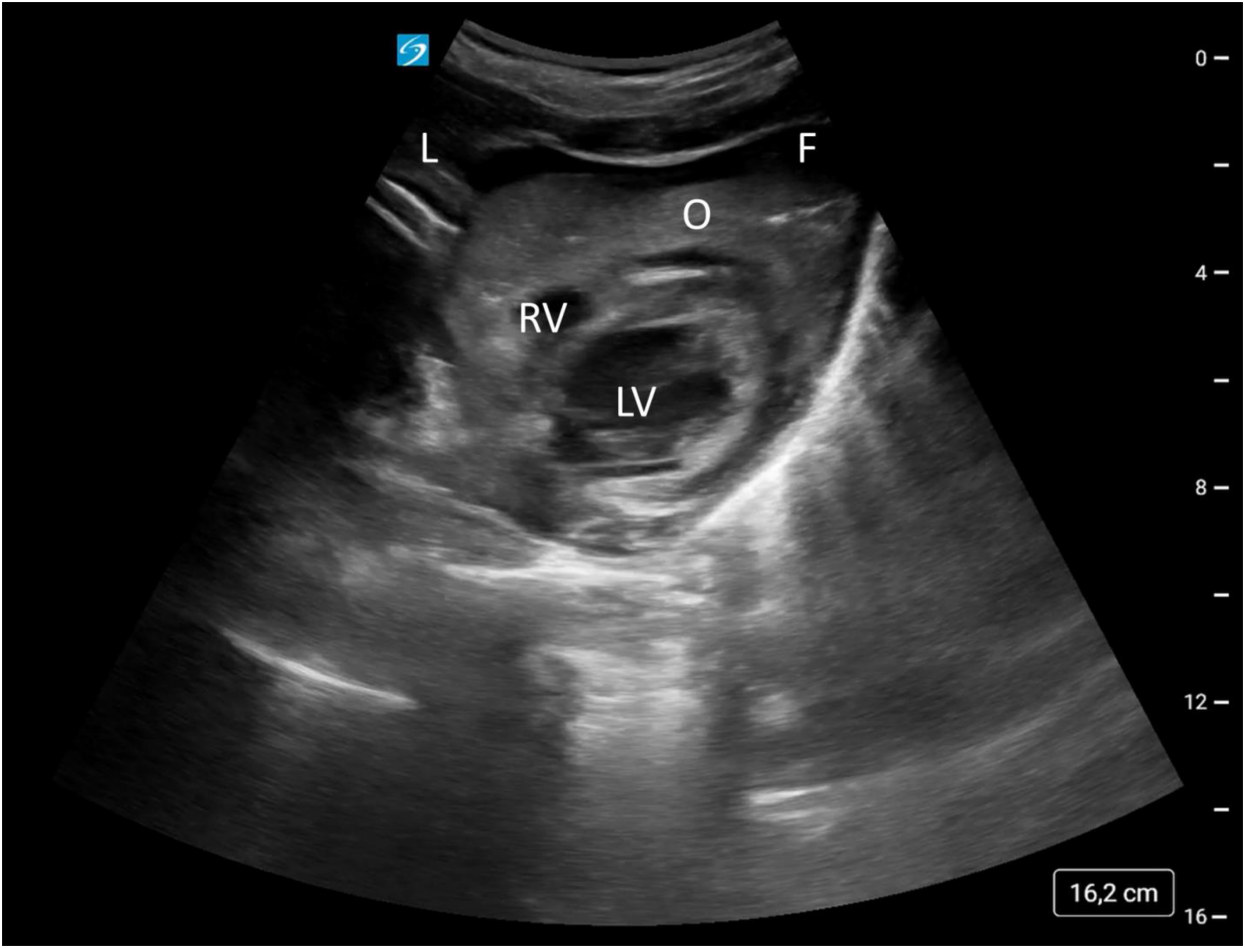
Subxiphoid view of the heart. L, liver; O, organized pericardial hematoma; F, fluid pericardial hematoma; LV, left ventricle; RV, right ventricle.

After skin suture, there was a drop in end-tidal CO_2_ from 40 to 21 mmHg, failure of pulse oximetry and arterial hypotension. Given the surgical procedure and the patient's medical history, the clinical examination initially suggested a tension pneumothorax or pulmonary embolism. After resuscitation was initiated, emergency ultrasonography was performed by a team member trained and certified in these diagnostics (2) resulting in the diagnosis of a pericardial tamponade with a partially organized und partially fluid hematoma (Figure 1, Supplementary Video S1).

Primary goal was to relief pericardial tamponade. As it was most likely a TCA, the indication for pericardiocentesis was weighed against clamshell thoracotomy. Because pericardiocentesis appeared to be faster, it was performed immediately using a commercially available Shaldon adult catheter by a team member trained in the technique of pericardiocentesis (3).

Subxiphoid pericardiocentesis was performed landmark-guided and assisted by sonographic positional control of the Seldinger wire, while chest compression was interrupted for this procedure. After placement of the wire, the Shaldon catheter was placed pericardially by Seldinger technique. After aspiration of 20 ml of blood through this Shaldon catheter, return of spontaneous circulation (ROSC) occurred immediately meaning 48 min after onset of TCA. Continuous venous blood could be aspirated via the now established pericardial drainage, suggesting a relevant cardiac perforation. The circulatory situation could be compensated with transfusion with 0-negative red blood cell concentrates. The cardiothoracic surgeons who have arrived in the meantime performed a median sternotomy with bandage scissors and cleared the remaining blood clots from the pericardium. The source of bleeding was an 8 mm diameter perforation on the basal posterior wall of the right ventricle close to the right coronary artery. The perforation was successfully sewn over with a felt-supported u-seam.

On the first postoperative day, sedation was terminated and the child was successfully extubated. With relevant clinical neurological changes compared to the preoperative condition, an MRI scan was performed which showed pathologic diffusion-weighted magnetic resonance imaging compatible with posthypoxic changes. An EEG performed on postoperative day six also showed a picture of posthypoxic encephalopathy.

In the following weeks, the neurological symptoms improved significantly. Four months later the child had learned to eat independently again, was interacting actively with his environment, and could respond adequately to questions.

## Discussion

The present case describes an in-hospital TCA in a child with satisfying neurologic outcome despite resuscitation duration of 48 min. It was the sonographic diagnosis that led to a relevant change in therapy and consecutive invasive emergency techniques.

Since 2015, the guidelines for cardiopulmonary resuscitation include an algorithm for the care of patients with TCA. Recommendations include treating reversible causes simultaneously, using sonography to identify the reversible cause, and relieving pericardial tamponade. Clamshell thoracotomy is one of the suggested options for this purpose, which was also considered in the present case but discarded for the benefit of the most likely faster pericardiocentesis. This approach is accompanied by The European Paediatric Advanced Life Support (EPALS) guidelines of 2021 especially if an emergency thoracotomy is not immediately feasible.

An argument against pericardiocentesis for treatment of a sanguineous pericardial tamponade is that the coagulated blood might clog the drainage. Accordingly Schneider et al. (3) in 2019 had considered pericardiocentesis for bloody pericardial tamponade to be unpromising. But in our case, 700 ml of blood could be aspirated with the inserted Shaldon catheter without problems until final surgical hemostasis, although sonographically coagulated blood was suspected. However, Schneider et al. (3) also considered pericardiocentesis as an option for temporary relief to allow time for appropriate therapy. There is uncertainty about the size of the drain to be inserted. If the internal lumen is smaller (for example, a 2 or 3 lumen central venous catheter), the drainage may be more likely to become blocked. Drains of similar dimensions to Shaldon-Katheter, such as a pigtail catheter (e.g., 8 or 10 French), could also be promising.

Our case shows that even a non-high risk intervention in children, such as a Hickman catheter implantation, can lead to TCA, which requires specific skills of all perioperative disciplines. A training in ultrasonographic diagnosis and rare invasive techniques help to apply this knowledge even in the rare case of TCA.

Regular interdisciplinary training in the recognition and treatment of complications is required to maintain and successfully implement this expertise.

## Summary

Immediate ultrasonographic diagnosis and invasive therapy of pericardial tamponade as cause of cardiac arrest determine neurological outcome. Resource reservation, training and experience, both in emergency ultrasonography and invasive emergency techniques, are important for this purpose. A commercially available Shaldon adult catheter is a considerable tool for emergency relief of even a sanguineous pericardial tamponade.

## Data Availability

The original contributions presented in the study are included in the article/**Supplementary Material**, further inquiries can be directed to the corresponding author.
